# Prophylactic inhibition of soluble epoxide hydrolase delays onset of nephritis and ameliorates kidney damage in NZB/W F1 mice

**DOI:** 10.1038/s41598-019-45299-5

**Published:** 2019-06-20

**Authors:** Jan Klocke, Arzu Ulu, Kaiyin Wu, Birgit Rudolph, Duska Dragun, Maik Gollasch, Wolf-Hagen Schunck, Bruce D. Hammock, Gabriela Riemekasten, Philipp Enghard

**Affiliations:** 1 Department of Nephrology and Intensive Care, Charité Universitätsmedizin Berlin, Berlin, Germany; 2 Department of Entomology and Nematology and Comprehensive Cancer Center, UC Davis, California, USA; 3 Department of Pathology, Charité Universitätsmedizin Berlin, Berlin, Germany; 4 Max-Delbrück-Centrum für Molekulare Medizin (MDC), Berlin, Germany; 5 Deparment of Rheumatology, Universitätsklinikum Schleswig-Holstein, Lübeck, Germany

**Keywords:** Experimental models of disease, Lupus nephritis, Lupus nephritis

## Abstract

Epoxy-fatty-acids (EpFAs), cytochrome P450 dependent arachidonic acid derivatives, have been suggested to have anti-inflammatory properties, though their effects on autoimmune diseases like systemic lupus erythematosus (SLE) have yet to be investigated. We assessed the influence of EpFAs and their metabolites in lupus prone NZB/W F1 mice by pharmacological inhibition of soluble epoxide hydrolase (sEH, EPHX2). The sEH inhibitor 1770 was administered to lupus prone NZB/W F1 mice in a prophylactic and a therapeutic setting. Prophylactic inhibition of sEH significantly improved survival and reduced proteinuria. By contrast, sEH inhibitor-treated nephritic mice had no survival benefit; however, histological changes were reduced when compared to controls. In humans, urinary EpFA levels were significantly different in 47 SLE patients when compared to 10 healthy controls. Gene expression of EPHX2 was significantly reduced in the kidneys of both NZB/W F1 mice and lupus nephritis (LN) patients. Correlation of EpFAs with SLE disease activity and reduced renal EPHX gene expression in LN suggest roles for these components in human disease.

## Introduction

Arachidonic acid (AA) has several pathways of metabolism. The cyclooxygenase (COX) dependent prostaglandins and the lipoxygenase (LOX) dependent leukotrienes are key variables in current treatment strategies concerning inflammation and pain; however, another group of cytochrome P450 (CYP) dependent AA derivatives, the epoxy fatty acids (EpFAs), are relatively uncharacterised.

Epoxyeicosatrienoic acids (EETs) are products of CYP epoxygenases, while other CYP enzymes with hydroxylase activity, and to some extent also LOX, lead to the production of hydroxyeicosatetraenoic acids (HETEs)^1^. EpFAs such as EETs are relatively short-lived and quickly metabolised by soluble epoxide hydrolase (sEH) and microsomal EH (mEH), to their less active diols, dihydroxyeicosatrienoic acids (DHETs). EETs are known to have anti-inflammatory and vasodilatory properties, which are opposed by the vasoconstriction and pro-inflammatory features of HETEs^2–4^. For a schematic overview of epoxide metabolism see Supplementary Fig. 1.

Manipulation of this anti-/proinflammatory equilibrium has been achieved by inhibition/knock-out of sEH and HETE-production with varying results. While an anti-inflammatory effect of EETs was suggested by reduced activation of NF-kB and consecutive downregulation of various pro-inflammatory cytokines and cell adhesion molecules, such as vascular cell adhesion protein 1 (VCAM1) on endothelial cells^5,6^, other studies also found an antihypertensive effect mediated by NO-release and enhanced natriuresis^1,7–9^. Further research hints at an improved outcome for cardiac^10,11^ and cerebral^12^ ischemia, and hypoxic pulmonary vasoconstriction^13^.

In the kidney, sEH inhibition has also been proposed as protective^14,15^. This effect might be due to an impairment of monocyte chemoattractant protein 1 (MCP-1) driven chemotaxis in the absence of DHETs^16^. However, other data indicate that sEH knock-out results in aggravated chronic and acute kidney insufficiency, due to locally increased HETE concentrations through a negative feedback loop^17,18^. Consistent with this, HETE inhibition led to ameliorated acute renal failure in a rat model^19^.

The broad efficacy in multiple disease models from increasing endogenous levels of EpFA, through blocking their metabolism by soluble epoxide hydrolase, has been difficult to explain^1^. A recent series of studies argued that an axis of mitochondrial dysfunction generating high reactive oxygen species acts through the endoplasmic reticulum stress pathway to initiate a variety of pathological outcomes. EETs, and thus sEH inhibitors (sEHI), seem to disrupt this chain of events, leading to a variety of illnesses including chronic pain and diabetes^20–23^.

The role of EpFAs in chronic inflammatory and autoimmune diseases like systemic lupus erythematosus (SLE) has yet to be investigated. In this study, we assessed the influence of EpFAs and their metabolites in human SLE and especially LN, by measuring serum and urine concentrations of a wide panel of EETs and similar epoxides and their metabolites, as well as HETEs (see Supplementary Fig. 1 for a full list of all measured metabolites). In addition, sEH inhibitor 1770 was administered to NZB/W F1 mice, in both a prophylactic and a therapeutic setting, to investigate the potential benefit of increasing certain bioactive lipids in lupus.

## Results

### EpFAs and sEH activity in the kidneys of lupus prone NZB/W F1 mice

**Figure 1:**
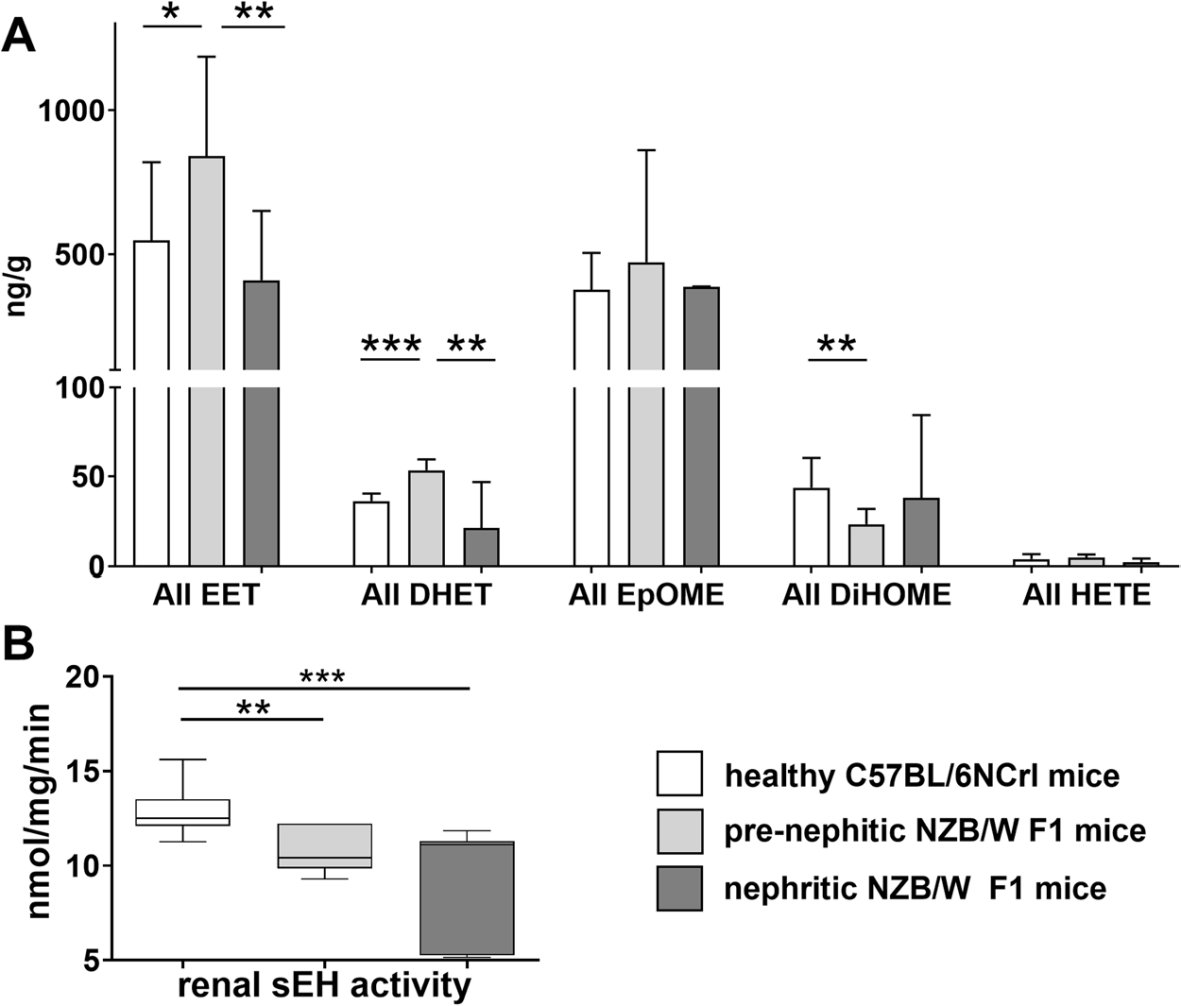
Renal epoxy fatty acids and metabolites in lupus prone NZB/W F1 and healthy C57BL/6NCRL mice. (**A**) Comparison of the cumulative renal EET (5,6-; 8,9-; 11,12-; 14,15-EET), EpOME (9,10-; 12,13-EpOME), DHET (5,6-; 8,9-; 11,12-; 14,15-DHET), DiHOME (9,10-; 12,13-DiHOME) and 20-HETE levels between healthy C57BL/6NCRL (n = 7), prenephritic (n = 6) and nephritic NZB/W F1 mice (n = 5). (**B**) Renal sEH activity in healthy C57BL/6NCRL (n = 12), prenephritic (n = 7) and nephritic NZB/W F1 mice (n = 7). Bars, median; boxplots, median and interquartile range; whiskers, range; white boxplot, healthy C57BL/6NCRL mice; light grey boxplot, prenephritic NZB/W F1 mice; grey boxplot, nephritic NZB/W F1 mice; *p < 0.05; **p < 0.01; ***p < 0.001. Values that passed the Kolmogorov-Smirnov test for Gaussian distribution were compared using the unpaired t test; otherwise, the Mann-Whitney test was applied.

To investigate the role of bioactive lipids in lupus, we analysed the concentrations of various CYP products and metabolites (see Supplementary Fig. 1 for a full list of metabolites) in the kidneys of lupus prone NZB/W F1 mice (n = 6, prenephritic NZB/W F1; n = 5, nephritic NZB/W F1) and the healthy C57BL/6NCRL (n = 7) mouse strain (cumulative data in Fig. 1; separated data in Supplementary Fig. 2). Kidneys of C57BL/6NCRL mice showed no significant change in any of the analysed metabolites with increasing age (n = 3 young versus n = 4 old; data not shown). For the NZB/W F1 comparison, young and old C57BL/6NCRL mice were presented as a combined cohort.

In the kidneys of NZB/W F1 mice before the onset of nephritis (prenephritic NZB/W F1), there was significantly higher levels for the sum of all EETs (p = 0.0388, 1.5-fold increase, Fig. 1A) than in healthy Bl6 mice. In nephritic mice, the levels of the sum of all EETs (p = 0.0087) as well as 14,15-EET (p = 0.0087) and 8,9-EET (p = 0.0087) were comparable to those for Bl6 controls, and significantly lower than in prenephritic NZB/W F1 mice. Similarly, epoxyoctadecenoic acids (EpOMEs) 8,9-EpOME (p = 0.0173) and 12,13-EpOME (p = 0.0087) concentrations in nephritic mice were lower when compared to prenephritic mice (Supplementary Fig. 2C). These changes between groups might at first glance indicate lower renal sEH activity in prenephritic mice.

Appropriately, a significantly lower sEH activity was found in the kidney of prenephritic NZB/W F1 mice (p = 0.0015), and even more so in nephritic mice (p = 0.0002), compared to Bl6 mice, as measured in an extended cohort (n = 7 prenephritic, n = 7 nephritic NZB/W F1, n = 12 Bl6; Fig. 1B). However, unexpectedly, the DHETs produced by sEH weren’t reduced in prenephritic mice, but were similarly enriched. The sum of all DHETs was higher (p = 0.0004) in prenephritic mice when compared to the C57BL/6NCRL controls and nephritic NZB/W F1 mice (p = 0.0087; Fig. 1A), mostly due to an increase in 5,6-DHET (Supplementary Fig. 2B). The sum of all sEH-produced dihydroxyoctadecenoic acids (DiHOMEs; p = 0.0097) was decreased in the prenephritic group.

### sEH Inhibition delayed onset of nephritis and improved survival in prenephritic NZB/W F1

**Figure 2:**
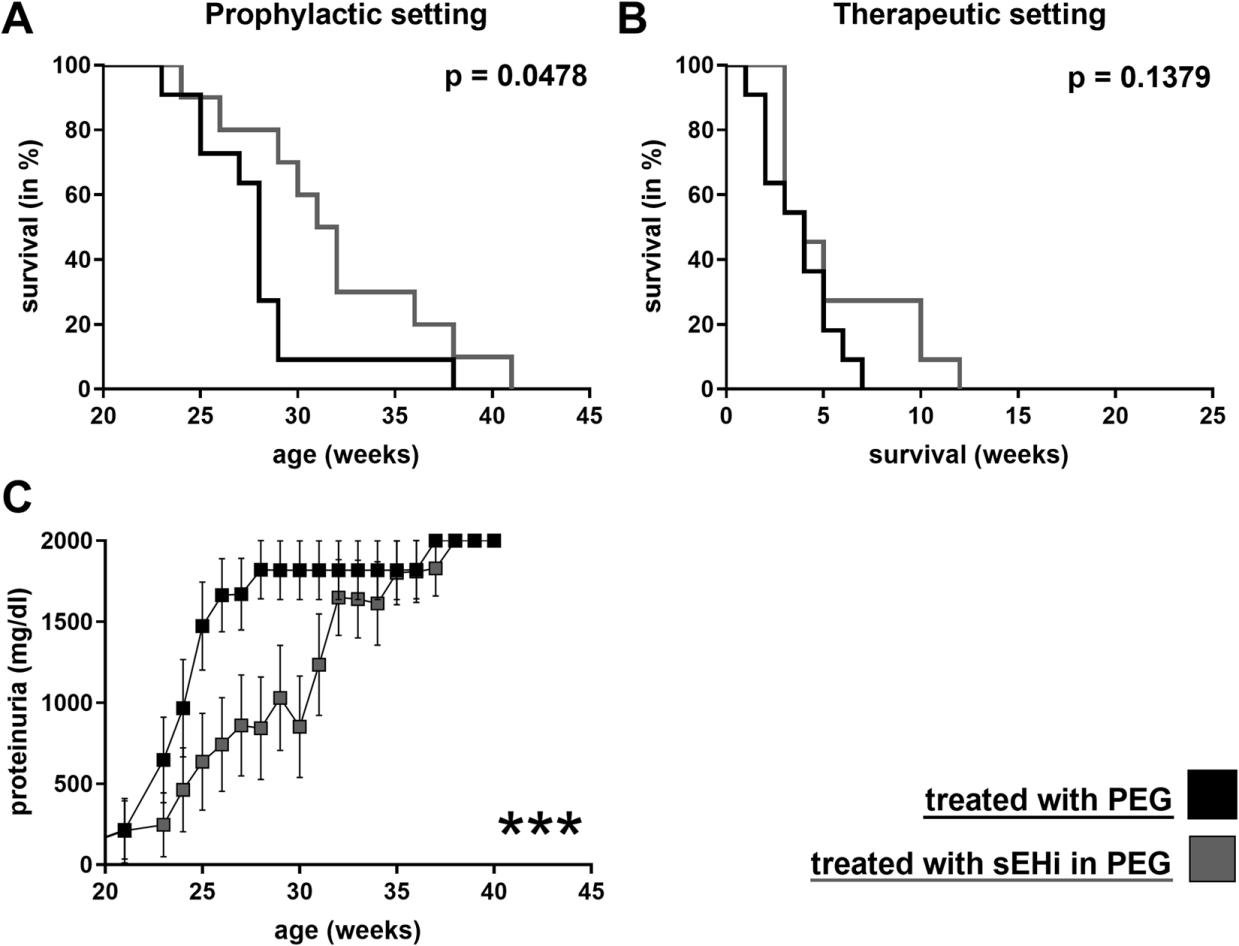
Impact of sEH inhibitor 1770 on the survival and proteinuria of NZB/W F1 mice. Survival of NZB/W F1 mice in a prophylactic (**A**) and a therapeutic (**B**) setting. Mice were treated with sEH inhibitor 1770 in 1% PEG400 (grey) or just 1% PEG400 (black). Each treatment included n = 11, except for prophylactic sEHI (n = 10); ***p < 0.001. Proteinuria comparison in the prophylactic setting. (**C**) Differences were measured using the log-rank test.

The sEH inhibitor 1770 (sEHI) was administered to NZB/W F1 mice to investigate the potential benefit of increased bioactive lipids in lupus. NZB/W F1 mice aged five months, without any signs of nephritis (no proteinuria), were either treated with sEHI or just the PEG co-solvent in their drinking water for 4 weeks (Fig. 2). Administration of sEHI in this prophylactic setting, before the onset of nephritis, significantly improved survival (n = 10 sEHI group, n = 11 PEG group; p = 0.0478 log-rank test). Consistent with the improved survival, reduced proteinuria was observed in the sEHI treated group (ANOVA p < 0.0001; Fig. 2A).

In a second clinical setting, sEHI was administered to mice with overt nephritis (proteinuria ≥ 300 mg/dl, median age 25.6 weeks) for 3 weeks, to test whether sEH inhibition was also able to influence established disease. Exposure was limited to 3 weeks, to avoid loss of animal due to the underlying lupus disease. In this therapeutic setting, no significant impact on survival was detectable (n = 11 in each group, experimental and control; p = 0.1379; Fig. 2B). In addition, no significant change in proteinuria was observed (data not shown).

At the beginning of treatment, prenephritic mice had significantly less anti-dsDNA-antibodies (mean 203 µg/ml) and proteinuria (mean 0 mg/dl) than nephritic mice (mean 360 µg/ml and 300 mg/dl, respectively; p < 0.0001). Treatment with sEHI had no significant influence on levels of dsDNA antibodies (data not shown).

### Treatment with sEHI ameliorated kidney damage

**Figure 3:**
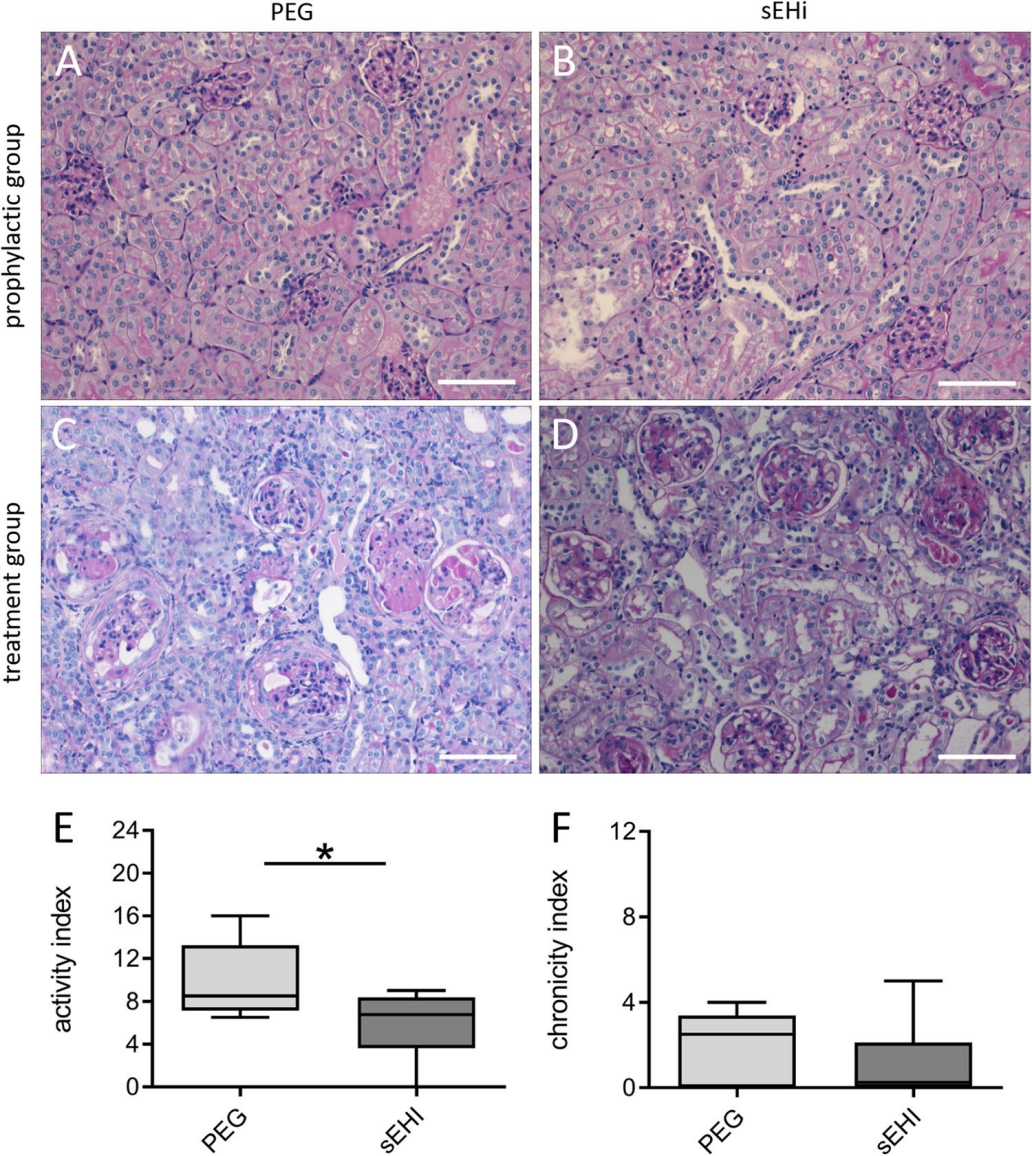
Impact of therapeutic sEH inhibition on renal histology findings in lupus prone NZB/W F1 mice. Representative kidney histology in the prophylactic group after four weeks of exposure to PEG (**A**) or sEHI (**B**; HE staining, 200x magnification). (**C**) Example histology from the treatment group after three weeks of exposure to PEG. Note the destroyed glomeruli (PAS staining, 200x magnification). (**D**) Kidney sample after 3-weeks exposure to sEHI, showing less severe glomerular changes than for the PEG group (PAS staining, 200x magnification). Scale bars = 100 µm. Comparison of the activity (**E**) and chronicity (**F**) indices of kidneys after three weeks of sEHI (n = 8) and PEG treatment (n = 8) of mice in the therapeutic setting. Boxplots show medians and interquartile ranges; whiskers, range; light grey, PEG treatment; dark grey, sEHI treatment; *p < 0.05. Compared using Mann Whitney test.

Kidney samples were analysed for severity of nephritis, to assess the impact of sEH-inhibition on kidney damage. Prenephritic mice were sacrificed after four weeks of exposure to either sEHI or PEG (Fig. 3). As expected, no histological differences were observed between the sEHI and PEG groups (n = 5 in each group; Fig. 3A,B). In nephritic mice, typical histological features of proliferative Lupus nephritis were obvious (Fig. 3C,D), and were scored using the Austin activity and chronicity index^24^. In nephritic mice treated for three weeks with sEHI, ameliorated kidney damage was observed in the form of a significantly lower activity index compared to the control group (p = 0.0391; Fig. 3E), while the index for chronic changes was unchanged between the two groups (n = 8 in each group; Fig. 3F). Of the histologic features assessed, a significant reduction in cellular crescents (p = 0.0451) and hyaline deposits (p = 0.0015) was found (data not shown).

### sEH inhibition induced significant changes in the renal bioactive lipid profile

**Figure 4:**
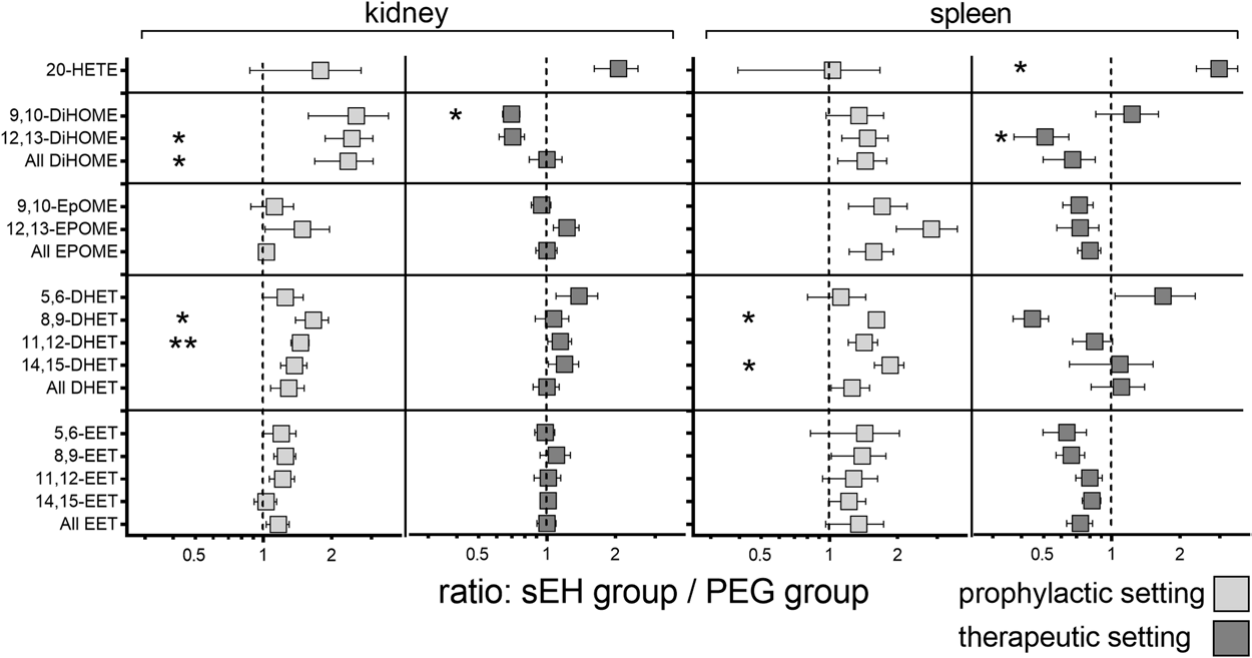
Comparison of metabolic changes in sEHI- and PEG-treated NZB/W F1 mice in the kidneys and spleen. To visualise changes due to sEH inhibition, the values are displayed as ratios (sEHI group/PEG group); consequently, a ratio >1 indicates an increased amount of that metabolite, while a ratio <1 indicates a decreased amount, compared to the PEG group. Significant differences between groups are measured by Mann Whitney test and are indicated by asterisks. Light grey, prophylactic setting (4 weeks sEHI exposure before onset of proteinuria); dark grey, therapeutic setting (3 weeks sEHI exposure after onset of proteinuria); *p < 0.05; **p < 0.01.

To monitor both systemic and renal changes, a broad panel of bioactive lipids (see Supplementary Fig. 1) were assayed in the kidneys and spleens of NZB/W F1 mice exposed to sEHI in the prophylactic and therapeutic setting (Fig. 4). While there was no direct change in the epoxide fatty acids (EETs and EpOMES) between the treatment and control groups, the administration of sEHI significantly altered the concentrations of their respective diol-metabolites (DHETs and DiHOMEs). In the spleens of prophylactically treated mice, sEHI significantly increased the levels of 14,15-DHET (p = 0.0240) and 8,9-DHET (p = 0.0430; n = 5 in each group). In the kidneys, sEH inhibition in the prophylactic group increased levels of 11,12-DHET (p = 0.006), 8,9- DHET (p = 0.0360), 12,13-DiHOME (p = 0.0170) and overall DiHOME (p = 0.0500; n = 10 sEHi group, n = 11 PEG group).

By contrast, in a therapeutic setting, NZB/W F1 mice treated with sEHI showed a decrease in renal 9,10-DiHOME (p = 0.0150; n = 8 in each group) and splenic 12,13-DiHOME (p = 0.0230; n = 3 in each group) compared to the control group. A significant increase in splenic levels of 20-HETE also occurred in the therapeutic setting (p = 0.0300).

To further investigate the effect of the altered bioactive lipid profiles on immune responses, a panel of cytokines, interleukin (IL)-1 $\alpha$, IL-2, IL-5, IL-21, IL-6, IL-10, interferon gamma (IFN $\gamma )$, tumor necrosis factor alpha (TNF $\alpha )$, IL-4, IL-17 and granulocyte-macrophage colony-stimulating factor (GM-CSF) were measured in plasma for the prophylactic and the therapeutic settings. In addition, spleens were removed and analysed as a major immunologic organ, where dysregulation of several immune subsets can be monitored. Splenic T cell subsets, B cells and plasma cells of prenephritic mice were monitored by flow cytometry after four weeks of treatment with either sEHi or PEG (Supplementary Fig. 3). Regarding cytokines, only GM-CSF levels were significantly decreased by prophylactic sEH inhibition (p = 0.0208; n = 5 for each group; Supplementary Fig. 3A), when compared to the control group. The increase in IL-5, IL-6, IFNg and TNFa between prophylactic and therapeutic groups was attributed to a general increase in inflammation due to a prolonged and unaltered course of disease. sEH inhibition did not significantly alter the monitored lymphocyte subsets (Supplementary Fig. 3B,C).

### Differences in urinary EpFAs between SLE patients and healthy controls

**Figure 5:**
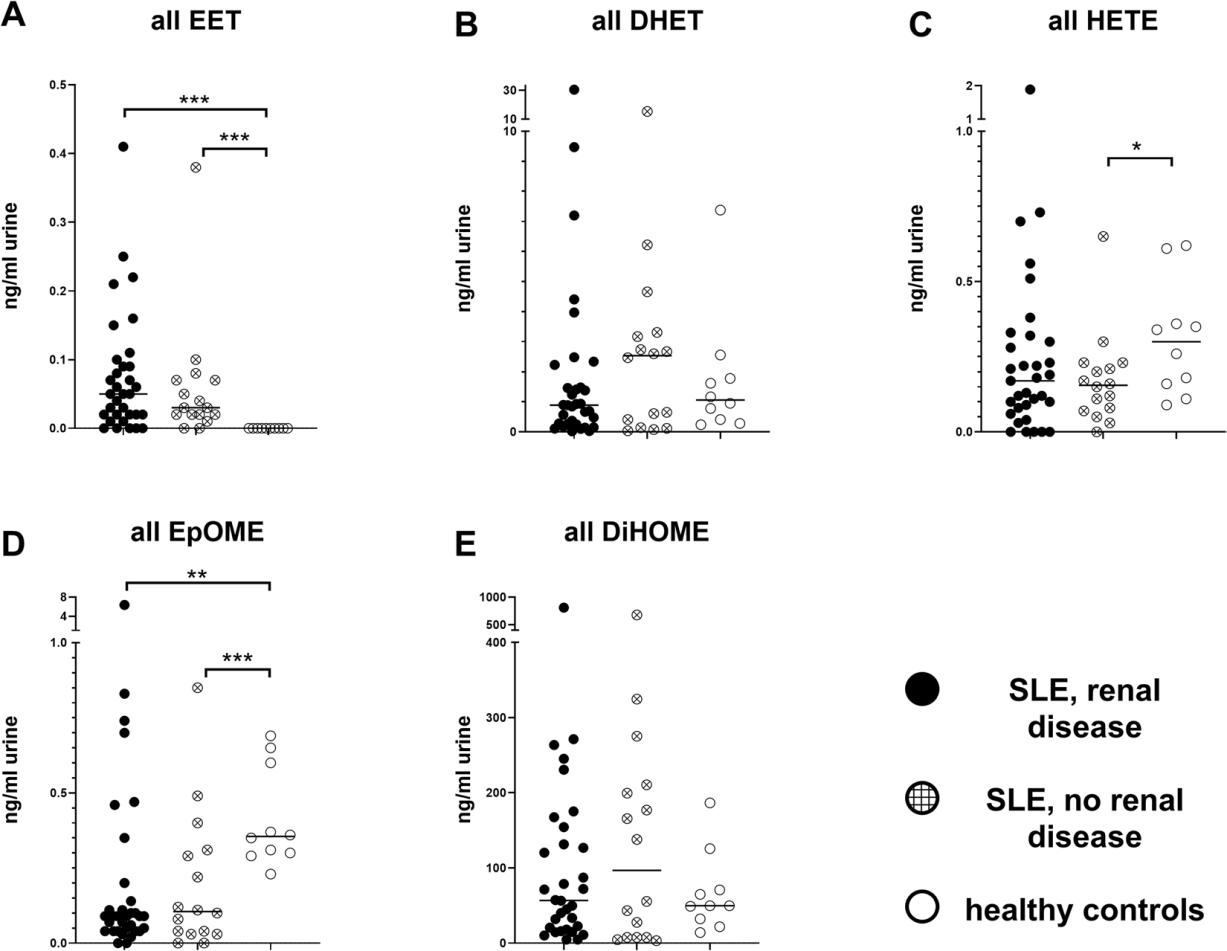
Urinary EpFA levels and metabolites in SLE patients and healthy controls. Bars, median; each dot represents one patient/one measurement; filled dots, LN patients; cross-hatched dots, SLE patients without renal disease; open dots, healthy controls. For a full list of all EpFAs measured in each group, see Supplementary Fig. 1 and Supplementary Table 1. *p < 0.05; **p < 0.01; ***p < 0.001.

In order to assess the potential implication of EpFAs and related bioactive lipids in SLE, the concentrations of lipid mediators (see Supplementary Fig. 1) were determined in serum and urine samples of 49 SLE patients (33 patients with acute or prior LN, and 16 patients without renal disease; Table 1). In addition, levels of urinary EpFAs were measured in 10 healthy controls. The bioactive lipids were not significantly different between the SLE disease groups in either urine (Fig. 5) or serum (data not shown), although patients with renal disease showed some positive correlations between disease activity based on the Systemic Lupus Erythematosus Disease Activity Index (SLEDAI) and various urinary and serum EpFAs (data not shown).

**Table 1:** Patient characteristics.

	SLE, LN	SLE, no LN	Healthy controls
n=	33	16	10
Female (%)	25 (76)	14 (88)	8 (80)
Age	37 (21─54)	48 (22─71)	31 (23─65)
SLEDAI	9 (0─25)	5 (0─15)	—
Creatinine (mg/dl)	0.92 (0.47─2.01)	0.81 (0.63─0.97)	0.75 (0.58─0.83)
Proteinuria (mg/d)	1799 (142─6724)	127 (52─165)	—

Values are means (range); LN, patients with current or former lupus nephritis; SLE, systemic lupus erythematosus; SLEDAI, SLE disease activity index.

By contrast, SLE patients had an altered urinary EpFA profile in comparison with healthy controls. In all SLE patients, urinary EET concentrations were already low; however, in the control group, EETs could not be detected at all (p < 0.0001; Fig. 5A). Paradoxically, the closely related epoxy lipids EpOMEs had slightly higher concentrations in the urine of healthy controls (p < 0.0001; Fig. 5D). In comparison, urinary concentrations of sEH produced dihydroxy lipids DHET and DiHOME were overall more pronounced, but did not differ between disease and control mice (Fig. 5B,E). Urinary 20-HETE tended to have a slightly higher concentration in healthy controls, albeit only when compared to SLE patients without renal disease (p = 0.0451; Fig. 5C).

### Renal EPHX2 gene expression was reduced in NZB/W F1 mice and LN patients

**Figure 6:**
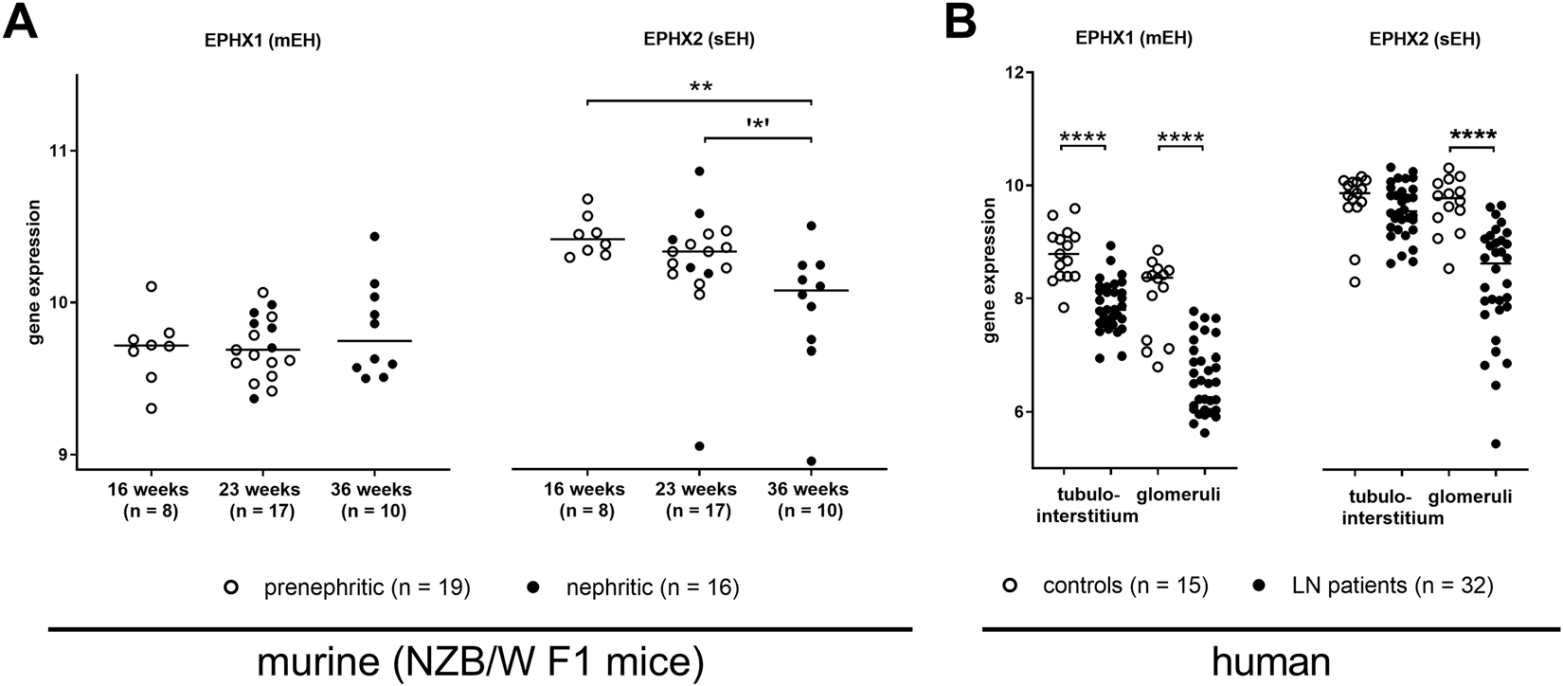
Renal mEH (EPHX1) and sEH (EPHX2) gene expression in NZB/W F1 mice and human LN patients and controls. (**A**) Renal EPHX gene expression in NZB/W F1 mice with increasing age and disease. Bars, median; every circle represents one value; filled circles, nephritic mice; open circles, prenephritic mice, *p < 0.05 (when comparing n = 11 prenephritic 23 week old mice with n = 10 nephritic 36 week old mice), **p < 0.01. (**B**) Comparison of renal EPHX expression in the tubulointerstitium and glomeruli of 32 patients with acute LN and 15 healthy pre-transplant donors. Bars, median; every circle represents one patient/value; filled circles, LN patients; open circles, controls; ****p < 0.0001. Values that passed the Kolmogorov-Smirnov test for Gaussian distribution were compared using the unpaired t test; otherwise, the Mann-Whitney test was applied.

We reviewed gene transcription data provided by Berthier *et al*. (GSE32591) via the Gene Expression Omnibus (GEO) database^25^. The dataset contained renal gene expression profiles for 35 NZB/W F1 mice (GSE32583), 32 human LN patients and 15 living transplant donors as controls (GSE32591). The murine dataset contained NZB/W F1 mice of varying age (16 weeks n = 8, 23 weeks n = 17, 36 weeks n = 10) and disease stage (prenephritic n = 19, nephritic n = 16). Older mice were generally sicker, with some 23 week-old and all 36 week-old mice being nephritic. Both age and disease status seemed to affect EPHX2 gene expression (encoding for sEH), with young mice having higher gene expression than old mice (p = 0.0014), and prenephritic mice showing higher expression than nephritic mice (p = 0.0263; Fig. 6A). By contrast, EPHX1 expression (encoding for mEH) was not dependent on age or disease stage in NZB/W F1 mice.

In the human dataset, we also compared EPHX1 and EPHX2 gene expression between LN patients and controls. Gene expression data were divided into interstitium and glomerular renal compartments (Fig. 6B). EPHX1 expression was significantly reduced in LN patients in both renal compartments (p < 0.0001). While there was no difference in EPHX2 gene expression in the tubulointerstitium, in the glomeruli it was significantly reduced in acute LN patients when compared to healthy controls (p < 0.0001).

In addition, gene expression of EpFA producing CYP epoxygenases CYP2C19, CYP2C8, CYP2C9 and CYP2J2 were examined in the same human dataset (Supplementary Fig. 4). CYP2C8 (p = 0.0203) and CYP2C9 (p < 0.0001) showed decreased tubulointerstitial gene activity in LN patients, thus potentially mirroring the decreased EET production in the murine model. CYP2C19 and CYP2J2 showed no difference between groups.

## Discussion

In this study, we showed the impact of altering the bioactive lipid profile in lupus prone NZB/W F1 mice by inhibition of sEH, and provided the first evidence that EpFAs and their metabolites might contribute to pathogenesis of human lupus nephritis. We collected observational data on three distinct levels (murine plasma and organ samples, human urine samples, murine and human renal gene expression), which all indicated a dysregulation in EpFA production and metabolisation in SLE and LN, although the results were not always consistent with the current understanding of EpFA metabolisation.

In the murine model (Fig. 1), a lower sEH activity was predictably associated with significantly elevated EET/EpOME concentrations; however, the concurrent elevation of corresponding diol-metabolites DHET was not anticipated. This occurred in prenephritic NZB/W F1 mice only, and might indicate increased CYP-dependent EET production that could not be sustained as the disease progressed. Why alterations in lipid profile were more pronounced in this early phase of the disease remained unclear. Human urine samples (Fig. 5) substantiated the murine findings with EETs exclusively being detected in SLE patients, albeit with a paradoxical elevation of EpOME concentrations in healthy controls.

Our survey of EPHX gene expression indicated renal downregulation of sEH with progressing disease in NZB/W F1 mice (Fig. 6A), and thus underlined the findings concerning renal sEH activity (Fig. 1B). Reduced sEH gene expression was also found in humans (Fig. 6B), where lower mEH and CYP epoxidase gene expression was observed in LN patients. The generally lower gene expression and sEH activity in lupus patients found in the current study might be explained by an accumulation of damaged tissue due to prolonged inflammation. Even so, overall, it remained unclear why inflammatory renal disease seemed to be associated with an intrinsic increase in anti-inflammatory lipids.

As our interventional data implied, altering the EpFA-signature in the prenephritic lupus prone mice by inhibition of sEH led to a significant improvement of survival and a transient reduction of proteinuria (Fig. 2). This was supported by the lack of histological changes in the kidneys of these mice (Fig. 3). The same could not be achieved in NZB/W F1 mice that already displayed signs of nephritis. While the lack of a benefit in overall survival in these mice was disappointing from a clinical perspective, the histological findings at least suggested an amelioration of kidney damage; therefore, sEHI might still improve the overall disease course. These findings were consistent with previous reports on the effect of sEHI on kidney damage, ranging from diabetic nephropathy^26^ to obstructive nephropathy^27,28^. More positive effects have recently been achieved by EET analogues in other disease models^29–32^. The improved outcome was mostly attributed to antihypertensive properties of the EETs; however, changes in inflammatory responses were also reported, such as reduced infiltration of neutrophils and macrophages^28,32^ and decreased interstitial fibrosis^27^.

To evaluate how sEH inhibition altered disease course in the current study, it was monitored on multiple levels. First, we observed changes in the profile of the bioactive lipids in both kidney and spleen. Interestingly, we detected no change in epoxy- fatty acids (EETs and EpOMEs), but only in their diol-metabolites (DHETs and DiHOMEs). We assumed that inhibition of sEH would lead to a reduction of DHETs and DiHOMEs, in comparison to the control group; however, this was only the case in the therapeutic setting, whereas prophylactically treated mice had significantly higher diol- concentrations than the controls. In the prophylactic setting, mice were treated over a longer timespan than their therapeutic counterparts, which might possibly have led to the activation of alternate metabolism pathways, such as mEH.

We also detected increased concentrations of the pro-inflammatory 20-HETE. Increased HETE after sEH-knockout has been attributed to a general shift in the metabolism of AAs towards the 20-HETE producing CYP-dependent pathway or the lipoxygenase pathways, which produce most other HETEs^17–19^. However, in this case it seemed more likely that the increased 20-HETE levels in the spleen were a result of prolonged inflammation, not sEH inhibition, as they were only detected in the therapeutic setting.

No significant changes in the initial immune response after sEHI treatment were found on measurement of cytokines, except for an increased GM-CSF concentration. However, due to the exploratory nature of the current study no correction for multiple testing was done. Consequently, in the absence of any other immunological changes, the validity of these findings was unclear. Cytokines were only detected in plasma; therefore, there might be renal changes that were not apparent in our data. The same conclusions could also be made for our monitoring of cellular immune responses, where we did not see any differences in splenic T cell and B cell subsets between groups.

Pharmacological inhibition of sEH with sEHI 1770 improved survival and proteinuria of lupus prone NZB/W F1 mice in a prophylactic treatment setting. Although the inhibition altered the bioactive lipid profile, the underlying immunologic mechanisms have yet to be determined. Reduced renal sEH gene expression in both murine and human settings, as well as alterations in the urinary lipid profile of SLE patients compared to healthy controls, suggested a role of bioactive lipids in human lupus disease.

## Methods

### Animals

C57BL/6NCRL and lupus prone NZB/W F1 (NZB × NZW F1) mice were obtained from the Bundesinstitut für Risikobewertung in Marienfelde, Berlin, Germany. Female mice were kept in a specific pathogen-free environment at the German Rheumatology Research Centre (DRFZ, Berlin, Germany. Standard chow and water were offered *ad libitum*. Experiments were performed in accordance with National Institutes of Health Guidelines on the Use of Laboratory Animals. The Animal Care Committee of the Charité University, Berlin approved the experiments (approval number G0003/11).

### Determination of sEH activities

Renal cytosolic fractions were prepared as described previously^33^. The assay was performed at 37 °C for 20 min in a final volume of 100 μL 0.1 M potassium phosphate buffer pH 7.2 containing 50 μM 14,15-EET as substrate. The reactions were started by adding the cytosolic fraction (3.5 μg of protein), and were terminated with 300 μl ethyl acetate. The remaining substrate and its product (14,15-DHET) were extracted and analysed by reversed-phase high performance liquid chromatography (RP-HPLC)^33^.

### Measurement of splenic and renal EpFA concentrations and renal sEH activity

Splenic and renal EpFA concentrations were measured with a Lipidomix kit (Lipidomix GmbH, Berlin, Germany) in healthy C57BL/6NCRL mice aged 2 months and 14 months, as well as in prenephritic (aged 2 months) and nephritic (aged 6 months) lupus prone NZB/W F1 mice. Tissue samples were homogenised in liquid nitrogen and aliquots corresponding to 30 mg wet weight were used for oxylipin analysis.

### Sample preparation and analysis of polyunsaturated fatty acid-derived lipid mediators and metabolites

Homogenised tissues from the animal experiments were spiked with an internal standard containing 10 ng of each of 14,15-EET-d8, 14,15-DHET-d11, 15-HETE-d8, 20-HETE-d6, LTB4-d4 and PGE2-d2 (Cayman Chemical, Ann Arbor, USA). In addition, 500 µl methanol and 300 µl 10 M sodium hydroxide solution was added to the tissue and it was shaken vigorously and heated for 30 min at 60 °C for alkaline hydrolysis. The samples were then brought to pH 6 with 500 µl 1 M sodium acetate buffer and acetic acid. After centrifugation, the supernatant was added to a Bond Elute Certify II column (Agilent Technologies, Santa Clara, USA) for solid phase extraction (SPE). The columns were preconditioned with 3 ml methanol, followed by 3 ml 0.1 mol/l sodium acetate buffer containing 5% methanol (pH 6). The SPE-columns were then washed with 3 ml methanol/H_2_O (50:50, vol:vol). For elution 2 ml n-hexane:ethyl acetate (25:75) with 1% acetic acid was used. The extraction was performed with a SPE vacuum manifold. The eluate was evaporated on a heating block at 40 °C under a stream of nitrogen, to obtain a solid residue. The residues were dissolved in 70 µL acetonitrile and analysed using an Agilent 1200 HPLC system (Agilent Technologies, Santa Clara, USA) with binary pump, degasser, autosampler and column thermostat, with a Kinetex C-18, 2.1 × 150 mm, 2.6 µm column (Phenomenex, Aschaffenburg, Germany), using a solvent system of 0.1% aqueous formic acid and acetonitrile. The elution gradient was started with 5% acetonitrile, which was increased within 0.5 minutes to 55%, 14.5 minutes to 69%, 14.6 minutes to 95% and held there for 5.4 minutes. The flow rate was set at 0.3 ml/min, and the injection volume was 7.5 µl. The HPLC was coupled to an Agilent 6460 Triplequad mass spectrometer (Agilent Technologies, Santa Clara, USA), with an electrospray ionisation source. The source parameters were: drying gas, 250 °C/10 l/min; sheath gas, 400 °C/10 l/min; capillary voltage, 4500 V; nozzle voltage, 1500 V; and nebulizer pressure, 30 psi. Analysis was performed with multiple reaction monitoring in negative mode. For further details see Arnold *et al*.^34^.

### Treatment with sEHI 1770

Prenephritic and nephritic NZB/W F1 mice were provided with the 1-trifluoromethoxyphenyl-3-(1-propionylpiperidin-4-yl) urea (TPPU; sEHI 1770)^1,35^ dissolved in drinking water. NZB/W F1 mice aged five months, without any signs of nephritis (no proteinuria), were treated with sEHI orally (n = 10). The solution for dosing was generated by adding 3.5 mg TPPU dissolved in 5 ml PEG400 to 500 ml warm drinking water with stirring, to give a final concentration of 7 mg/l TPPU, equalling a dose of 1 mg/kg/day. Control animals were treated with the PEG400 solvent alone (n = 11). Treatment resulted in mean blood concentrations of 2.396 nM and 2.027 nM after one and two weeks of treatment, respectively, indicating TPPU was at near steady state levels. The concentrations were comparable to those in previous TPPU studies^36^. In a second experimental setting, oral administration of sEHI in PEG400 solvent (n = 11) or PEG400 alone (n = 11) was initiated in mice with overt nephritis (proteinuria ≥300 mg/dl, median age 25.6 weeks). Proteinuria was measured using Multistix reagent strips (Bayer Diagnostics, Germany).

### Histological analysis

Prenephritic mice were sacrificed after four weeks of exposure to PEG + sEHI or to PEG. Nephritic mice were treated for three weeks after the onset of nephritis with PEG + sEHI or PEG. In the prophylactic setting, mice were treated for four weeks before analysis; however, in the treatment setting the exposure was limited to three weeks, to avoid loss of animals due to the underlying lupus disease. Kidneys were removed and fixed with 2% paraformaldehyde. The samples were paraffin embedded, cut in sections and stained with haematoxylin/eosin (HE) and periodic acid-Schiff (PAS) reagents. Slides were examined by experienced pathologists blinded to the treatment conditions; typical histological features of LN were determined and scored using the Austin activity and chronicity index^24^.

### Additional analysis

Splenic cells and plasma cytokines were measured by flow cytometry and multiplex assays, respectively, in prenephritic and nephritic NZB/W F1 mice (Supplementary Fig. 3).

For flow cytometry, a cell suspension of freshly removed spleens (removed concurrently with the kidneys) was generated by dissipating and straining through a 70 µm cell strainer. Cells were permeabilised using the Fix & Perm Cell Permeabilization Kit (Invitrogen, Frederick, MD, USA). To block non-specific binding, cells were stained in phosphate-buffered saline with 1% bovine serum albumin containing 10% human immunoglobulin G (IgG; Flebogamma, Grifols, Langen, Germany). Staining was performed for 20 minutes at 4 °C with CD3-PE, CD8-Cy5, CD19-PB, CD44PB, (all DRFZ, Berlin, Germany), CD138-PE, Ki67-PECy7 (both BD, Franklin Lakes, New Jersey, USA), CD4-PerCP, CD69-FITC, and FoxP3-APC (all eBioscience, San Diego, California, USA). To exclude dead cells, phycoerythrin (DRFZ, Berlin, Germany) was added immediately before flow cytometry. The samples were measured using a MACS Quant Analyzer (Miltenyi Biotec GmbH, Bergisch Gladbach, Germany). After exclusion of doublets and dead cells, T cells were gated by CD3 expression and B cells were gated by CD19 expression. Further subtyping was achieved with the markers given above. Datasets were analysed using Flowjo Software (Tree Star, Ashland, Oregon, USA).

Additionally, a panel of cytokines (IL-1α, IL-2, IL-5, IL-21, IL-6, IL-10, IFN-γ, TNF-α, GM-CSF, IL-4 and IL-17) was assessed in thawed serum samples, stored at −80 °C. A Flowcytomix multiplex assay kit (eBioscience, San Diego, California, USA) was used according to the standard procedure suggested by the supplier.

### Patients

Serum and urine samples of 47 SLE patients and 10 healthy controls were analysed for a wide array of lipid mediators: 5,6-EET, 8,9-EET, 11,12-EET, 14,15-EET, 9,10-EpOME, 12,13-EpOME, 5,6-DHET, 8,9-DHET, 11,12-DHET, 14,15-DHET, 9,10-DiHOME, 12,13-DiHOME, 19-HETE, 20-HETE, and the sum of all EETs, EpOMEs, DHETs, DiHOMEs and HETEs. We collected and stored 49 serum and urine samples from 47 patients with SLE between 2009 and 2012. The samples were analysed in 2012. Two patients were evaluated twice with at least two years between measurements. Samples from healthy controls were collected and analysed in 2019.

SLE patients were divided into two groups based on their renal involvement, “SLE, LN” and “SLE, no LN.” The SLEDAI was calculated for all patients. Thirty-three patients had LN, as determined by at least one positive biopsy for current LN (n = 16) or during a prior course of the disease (n = 17). The remaining 16 patients had no diagnosed LN (see Table 1 for patient characteristics). Samples were collected from patients of the Departments of Rheumatology and Clinical Immunology, and Nephrology, Charité University Hospital, Berlin, Germany. The ethics committee of Charité University Hospital (Charité EA1/034/10) approved the study, and the investigation was carried out in compliance with the Declaration of Helsinki. Informed consent was obtained from all patients before participation.

### Serum and urinary EpFAs in SLE patients

Serum and urine samples were collected and immediately centrifuged, with the resulting supernatant being stored at −80 °C. Frozen samples were analysed as detailed above.

### Gene transcription data

The gene expression profile GSE32592 from the GEO database was downloaded^37^, and the gene expression of EPHX1 (mEH) and EPHX2 (sEH) was reviewed. In their original work on this dataset, Berthier *et al*. identified renal transcription features common in human LN and murine LN models^25^. The series contained gene transcription profiles of glomeruli and the tubulointerstitium of 32 LN patients and 15 controls represented by pre-transplant healthy living donors (GSE32591, for further patient information see Berthier *et al*.^25^). It also included profiles of 35 NZB/W F1 mice with varying ages and disease stages (GSE32583).

### Statistical analysis

Datasets that passed the Kolmogorov-Smirnov test for Gaussian distribution were compared using the unpaired t test/one way analysis of variance (ANOVA); otherwise, the Mann-Whitney test/Kruskal-Wallis test was applied. Significances between individual groups were calculated by t-test/Mann-Whitney test. For comparisons of three groups, those with statistically significant differences also passed the one way ANOVA or Kruskal-Wallis test. Correlations were calculated by Spearman correlation. Survival was calculated by log-rank test. All medians, means, ANOVA tests, Kruskal-Wallis tests, unpaired t-tests, Mann–Whitney tests, Spearman correlations and log-rank tests were calculated using GraphPad Prism 7 software (GraphPad Software, San Diego, California, USA). Due to the explorative nature of the study, we did not control for multiple comparisons.

### Ethical approval and consent to participate

Experiments were performed in accordance with the National Institutes of Health Guidelines on the Use of Laboratory Animals. The Animal Care Committee of Charité University Berlin approved the experiments (approval number G0003/11). The ethics committee of Charité University Hospital (Charité EA1/034/10) also approved the study, and the investigation was carried out in compliance with the Declaration of Helsinki. Informed consent was obtained from all patients before participation.

## Supplementary information

Supplementary information

## Data Availability

The datasets generated and/or analysed during the current study are available from the corresponding author by request.
