# Supplementary material for: Prophylactic inhibition of soluble epoxide hydrolase delays onset of nephritis and ameliorates kidney damage in NZB/W F1 mice
Source: Sci Rep. 2019 Jun 20;9:8993. doi: 10.1038/s41598-019-45299-5 (PMC6586931; doi:10.1038/s41598-019-45299-5)
Supplement: Supplementary file 1 — Supplementary information [file 41598_2019_45299_MOESM1_ESM.pdf]

Prophylactic inhibition of soluble epoxide hydrolase delays onset of nephritis and ameliorates kidney damage in NZB/W F1 mice

Jan Klocke[1], Arzu Ulu[2], Kaiyin Wu[3], Birgit Rudolph[3], Duska Dragun[1], Maik Gollasch[1], Wolf-Hagen Schunck[4], Bruce D. Hammock[2], Gabriela Riemekasten[5] and Philipp Enghard[1].

SUPPLEMENTARY MATERIAL

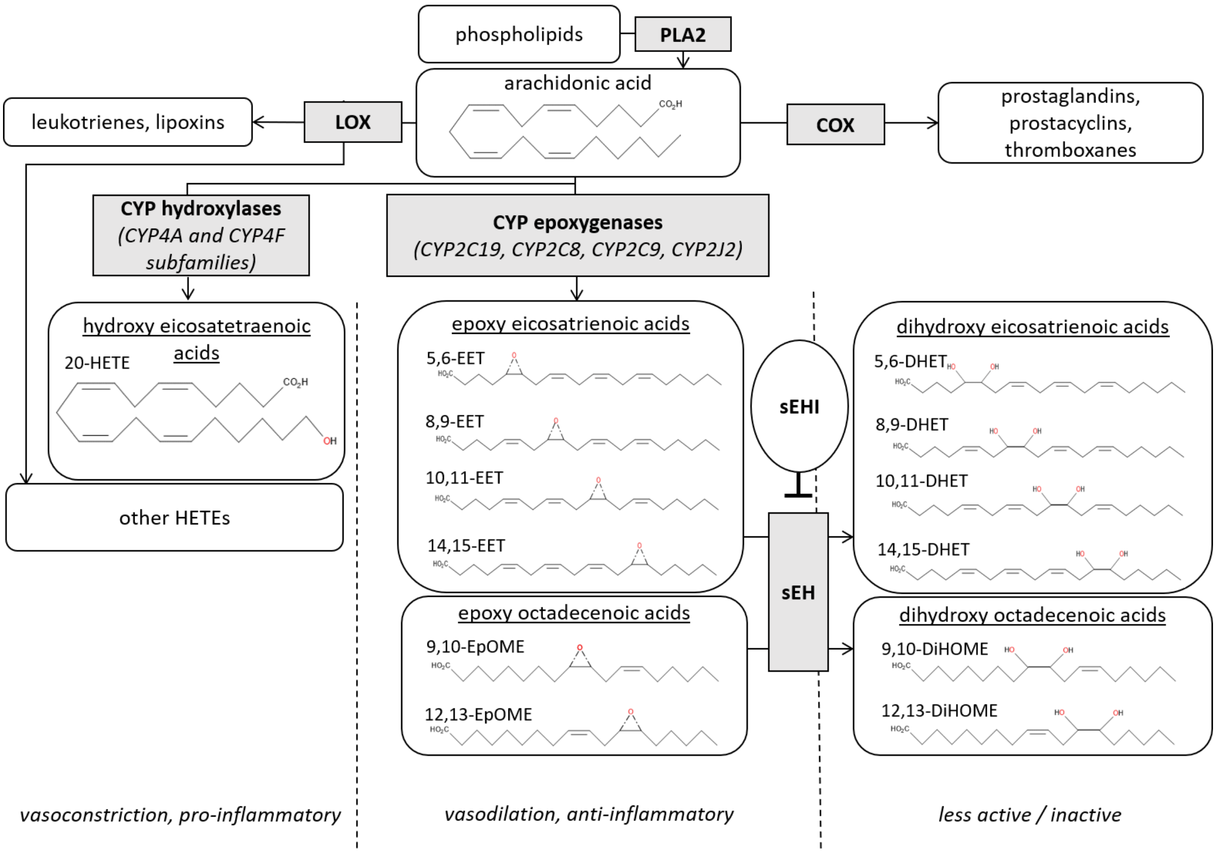

Supplementary Fig. 1 │ Metabolism of arachidonic acid derivatives and epoxy fatty acids. Arachidonic acids are metabolised by a range of cytochrome P450 enzymes (CYP) with either hydroxylase or epoxygenase activity. Epoxygenase products, epoxyeicosatrienoic acids (EET) and epoxyoctadecenoic acids (EpOME) are further metabolised by soluble epoxide hydrolase (sEH) to their diols, dihydroxyeicosatrienoic acids (DHET) and dihydroxyoctadecenoic acids (DiHOME). COX, cyclooxygenase; HETE, hydroxyeicosatetraenoic acids; LOX, lipoxygenase; PLA2, phospholipase A2; sEHI, sEH inhibitor.

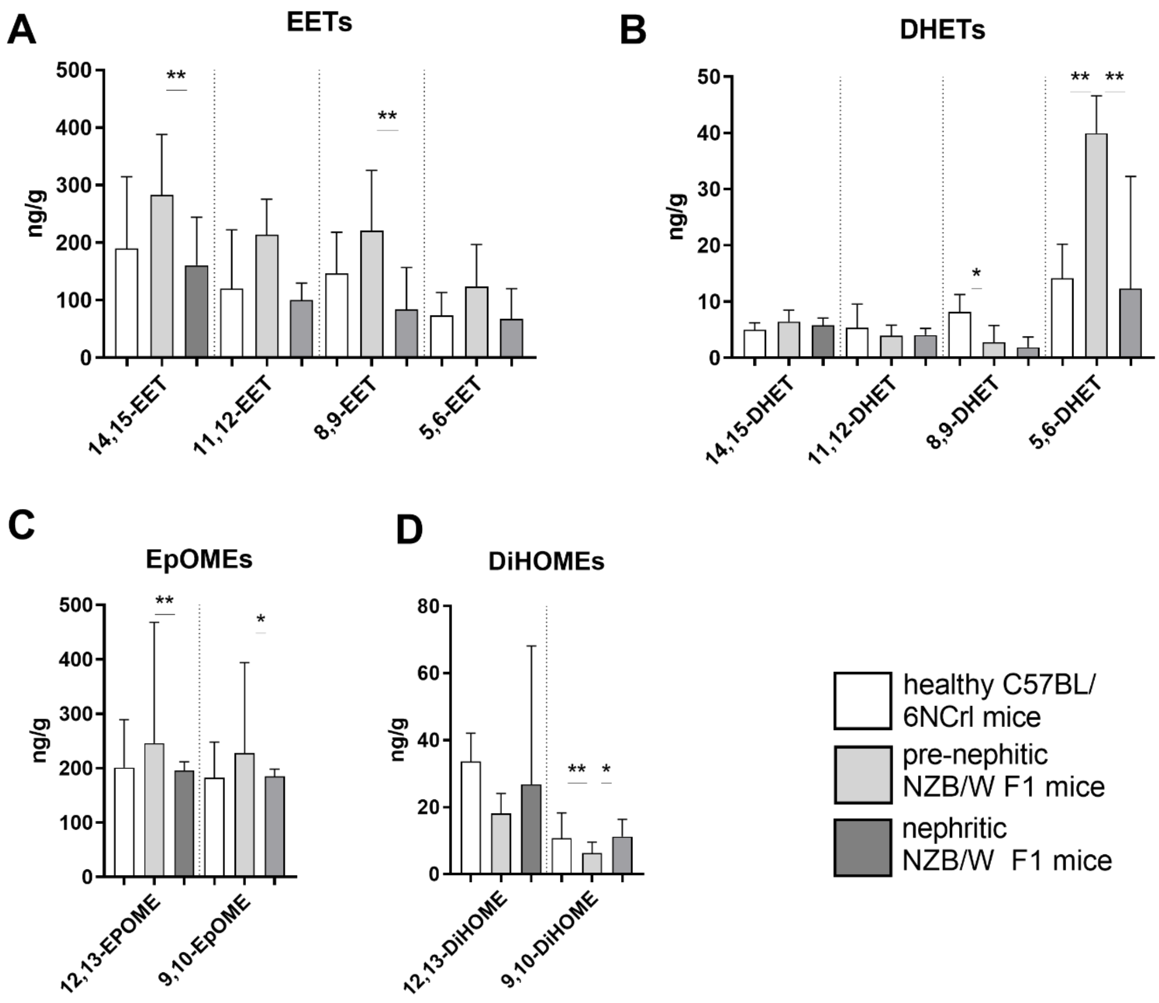

Supplementary Fig. 2 │Renal epoxy fatty acids and metabolites in lupus prone NZB/W F1 and healthy Bl6 mice. Comparison of levels of (**A**) single renal EETs (5,6-; 8,9-; 11,12-; 14,15-EET); (**B**) DHETs (5,6-; 8,9-; 11,12-; 14,15-DHET); (**C**) EpOMEs (9,10-; 12,13-EpOME); and (**D**) DiHOMEs (9,10-; 12,13-DiHOME). Bars, median; whiskers, range; white bar graph, healthy C57BL/6NCrL mice, n = 7; light grey bar graph, prenephritic NZB/W F1 mice, n = 6; grey bar graph, nephritic NZB/W F1 mice, n = 5; * $p < 0.05$; ** $p < 0.01$. Values were compared using the Mann-Whitney test.

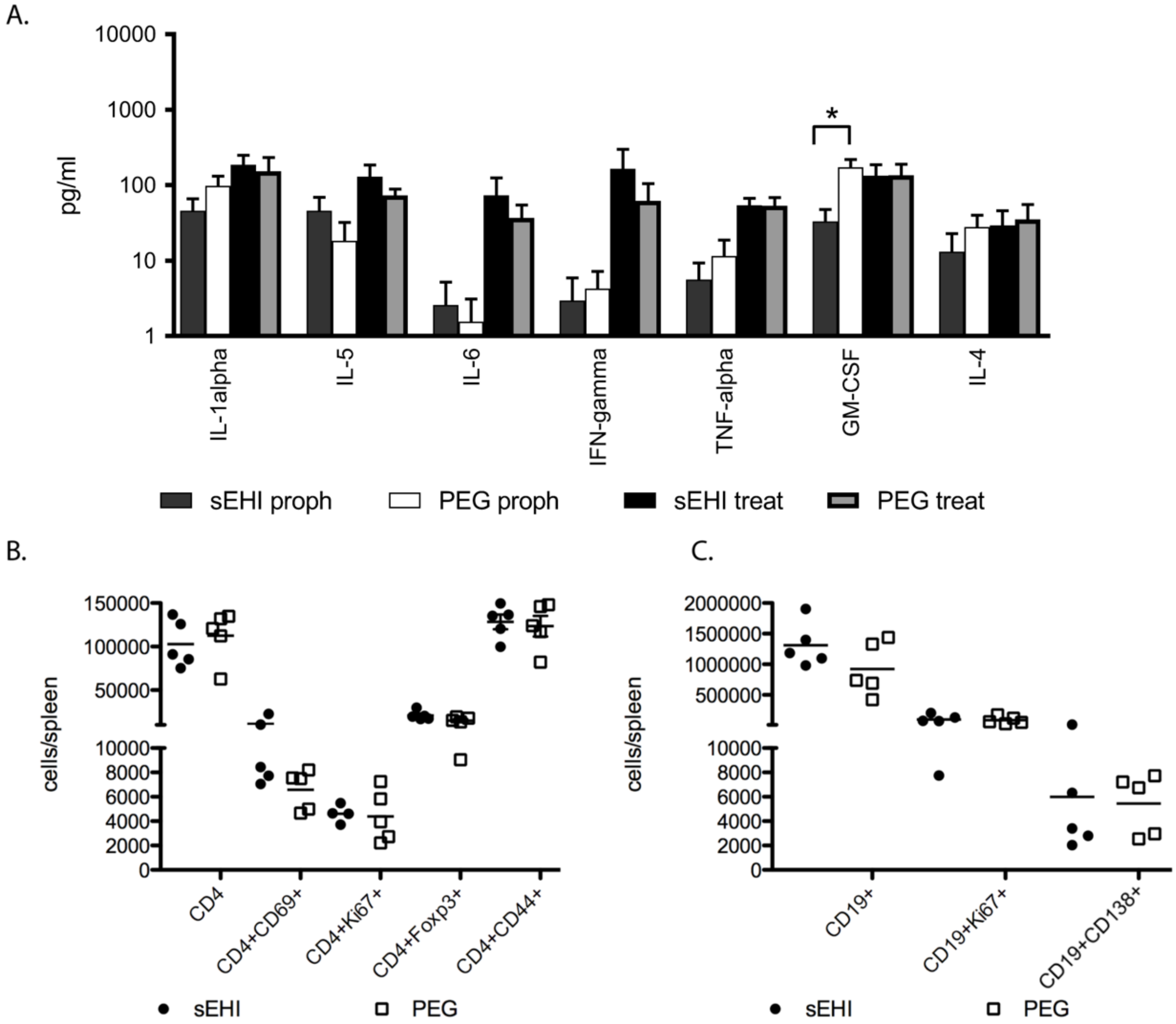

Supplementary Fig. 3 │ Influence of sEH-inhibition on the autoimmune process of NZB/W F1 mice. (**A**) Comparison of plasma cytokine levels. Displayed are the respective cytokine levels of sEHI and PEG treated mice in the prophylactic (proph) and treatment (treat) settings. Bar plot, mean; whiskers, maximum values; * $p < 0.05$. (**B**), (**C**) Unaltered splenic T- and B-cell subsets under prophylactic sEHI exposure. Dot plot: line, median; circle, sEHI treated mice, n = 5; square, controls, n = 5.

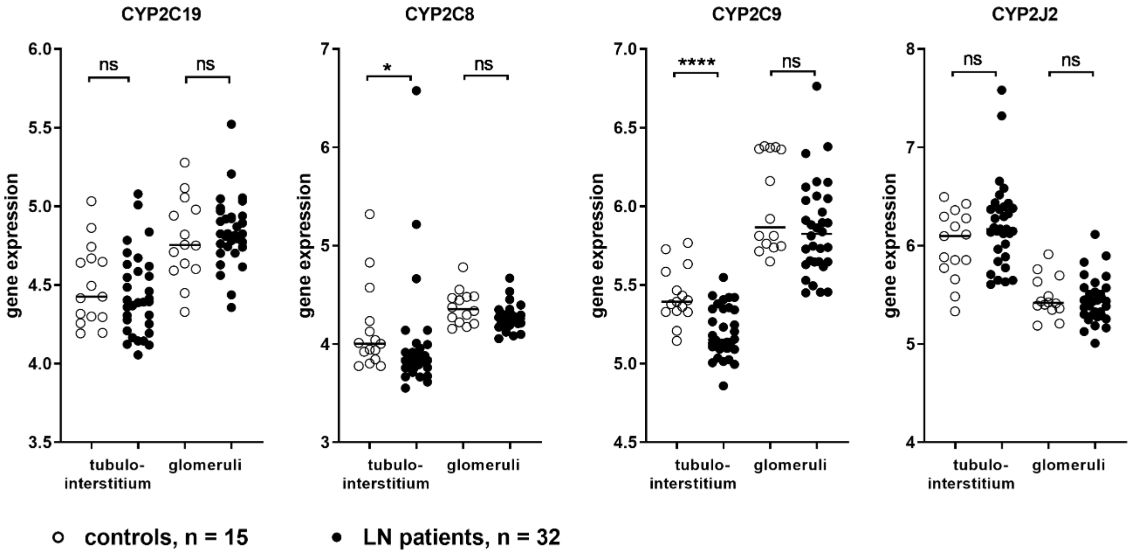

Supplementary Fig. 4 │ Gene expression of CYP epoxygenases in lupus nephritis patients and healthy controls. Comparison of renal CYP epoxygenase gene expression in the tubulointerstitium and glomeruli of 32 patients with acute lupus nephritis (LN) and 15 healthy pre-transplant donors. Bars, median; every circle represents one patient/value; filled circles, LN patients; open circles, controls; * $p < 0.05$; **** $p < 0.0001$. Values that passed the Kolmogorov-Smirnov test for Gaussian distribution were compared using the unpaired t test; otherwise, the Mann-Whitney test was applied.
